# Perceived rules and accessibility: measurement and mediating role in the association between parental education and vegetable and soft drink intake

**DOI:** 10.1186/s12937-016-0196-3

**Published:** 2016-08-17

**Authors:** Mekdes K. Gebremariam, Nanna Lien, Liv Elin Torheim, Lene F. Andersen, Elisabeth L. Melbye, Kari Glavin, Solveig E. S. Hausken, Ester F. C. Sleddens, Mona Bjelland

**Affiliations:** 1 Department of Nursing and Health Promotion, Faculty of Health Sciences, Oslo and Akershus University College of Applied Sciences, P.O. Box 4, Olavs Plass Street, 0130 Oslo, Norway; 2 Department of Nutrition, Faculty of Medicine, University of Oslo, P.O.Box 1046, Blindern, NO-0316, Oslo, Norway; 3 Norwegian School of Hotel Management, University of Stavanger, 4036 Stavanger, Norway; 4 Department of Nursing, Diakonova University College, Oslo, Norway; 5 Department of Health Promotion, School of Nutrition and Translational Research in Metabolism (NUTRIM), Maastricht University Medical Center, Maastricht, The Netherlands

**Keywords:** Soft drinks, Vegetables, Socioeconomic differences, Parental education, Mediation, Adolescents

## Abstract

**Background:**

The existence of socioeconomic differences in dietary behaviors is well documented. However, studies exploring the mechanisms behind these differences among adolescents using comprehensive and reliable measures of mediators are lacking. The aims of this study were (a) to assess the psychometric properties of new scales assessing the perceived rules and accessibility related to the consumption of vegetables and soft drinks and (b) to explore their mediating role in the association between parental education and the corresponding dietary behaviors.

**Methods:**

A cross-sectional survey including 440 adolescents from three counties in Norway (mean age 14.3 years (SD = 0.6)) was conducted using a web-based questionnaire. Principal component analysis, test-retest and internal reliability analysis were conducted. The mediating role of perceived accessibility and perceived rules in the association between parental education and the dietary behaviors was explored using linear regression analyses.

**Results:**

Factor analyses confirmed two separate subscales, named “accessibility” and “rules”, both for vegetables and soft drinks (factor loadings >0.60). The scales had good internal consistency reliability (0.70–0.87). The test–retest reliability of the scales was moderate to good (0.44–0.62). Parental education was inversely related to the consumption of soft drinks and positively related to the consumption of vegetables. Perceived accessibility and perceived rules related to soft drink consumption were found to mediate the association between parental education and soft drink consumption (47.5 and 8.5 % of total effect mediated). Accessibility of vegetables was found to mediate the association between parental education and the consumption of vegetables (51 % of total effect mediated).

**Conclusion:**

The new scales developed in this study are comprehensive and have adequate validity and reliability; they are therefore considered appropriate for use among 13–15 year-olds. Parents, in particular those with a low educational background, should be encouraged to increase the accessibility of vegetables and to decrease the accessibility of soft drinks, in particular during dinner. Enforcing parental rules limiting soft drink intake in families with low parental education also appears relevant.

## Background

Promoting healthy dietary behaviors is crucial to combat the obesity pandemic among youth [1]. In Norway, positive trends in the consumption of vegetables and soft drinks have been documented in the past decade; however a majority of adolescents still do not meet national dietary recommendations [2]. Efforts to improve dietary behaviors require knowledge about the factors influencing these behaviors. In this regard, several studies have explored the correlates of dietary behaviors among youth [3]. Home environmental factors such as availability and accessibility of food have consequently been identified as important correlates of the consumption of fruits and vegetables (FV) [4–7], as well as sugar-sweetened beverages [8, 9]. Parental rules have also been found to be associated with the consumption of unhealthy foods including soft drinks with sugar [10–14], although results appear to depend on the types of rules measured [10]. The association between rules and the consumption of healthy foods such as FV is on the other hand more equivocal. A systematic review found that family rules (i.e. parents demanding that FV be consumed or allowing that as much FV as wanted be consumed) were consistently positively associated with children’s FV consumption whereas such rules were found to be unrelated to fruit consumption among adolescents [4].

Despite the large volume of literature exploring the correlates of dietary behaviors, focus on the validity and reliability of the measures of correlates seems to be lacking [3, 15]. A high variation in the conceptualization and measurement of correlates is another challenge which makes comparability between studies difficult. Perceived accessibility of foods and parental rules can cover different dimensions which should be considered when developing measures, e.g. rules can include aspects related to when and how much food can be consumed. The measures should also have adequate psychometric properties tested among relevant groups in order to ensure the validity of results when these correlates are used.

There is consistent evidence regarding socioeconomic differences in dietary behaviors, with youth with a low socioeconomic position (SEP) having less favourable dietary behaviors [6, 16–19], including a lower consumption of vegetables and a higher consumption of sugar-sweetened beverages. However, the mechanisms behind these socioeconomic differences in dietary behaviors remain poorly understood. One explanation for these differences could be the variation in the correlates of dietary behaviors among different socioeconomic subgroups, as evidenced by a recent systematic review of the literature among 9–13 year-olds [16]. The review found that SEP was consistently positively related to nutrition knowledge, parental modelling as well as home food availability and accessibility; associations between SEP and parental feeding practices were indeterminate [16]. However, these differences do not reflect if and to what extent these correlates are responsible for socioeconomic differences in dietary behaviors. Such evidence is best obtained from studies assessing the mediating effect of correlates in the relationship between SEP and dietary behaviors. Identifying modifiable mediators would provide valuable information for interventions aimed at tackling socioeconomic differences in these behaviors. Accessibility and parental rules are two such important modifiable correlates of soft drink and vegetable intake [5–14] that can potentially mediate socioeconomic differences in these behaviors. However, only few mediation studies exploring socioeconomic differences in soft drink consumption [9, 20, 21] and in vegetable or FV consumption [19, 22, 23] are currently available. Based on these studies, accessibility has been identified as a mediator of socioeconomic differences in soft drink [9, 20, 21] and vegetable [19, 22] consumption. Parental rules were found to mediate socioeconomic differences in soft drink [9] and vegetable [23] consumption. However measures with single items were used in half of these studies [9, 20, 23], which makes it unlikely that all dimensions of food accessibility and parental rules were covered. In addition, all except one of these studies [21] included participants younger than those in the present study. The role of parental rules and home accessibility of foods might change with increasing age, in particular in the adolescence period which is marked by increasing autonomy [24]. There is thus a need for studies investigating the role of accessibility and parental rules in reducing socioeconomic differences in soft drink and vegetable consumption among adolescents using comprehensive and validated measures.

Against this background, this study aimed to explore the psychometric properties of new scales assessing adolescents’ perceived accessibility of and perceived rules related to the intake of vegetables and soft drinks. It also assessed whether these factors mediated the association between parental educational level and the consumption of vegetables and soft drinks.

## Methods

### Design and sample

The participants in this study are pupils from a convenience sample of five secondary schools in three counties (Oslo, Akershus, Vestfold) of Norway. In total, 1136 adolescents were invited to participate in this cross-sectional study and 440 adolescents (39 %) participated. Of these, 54 adolescents (26 % of the 208 invited) participated in a test-retest study. The test and retest were conducted 10–14 days apart. The adolescents were recruited at school using invitation letters sent to parents via teachers. Information about the study and consent forms were also included in the package to parents. The details about the methods are presented in a previous paper [25].

### Data collection and measures

A web-based questionnaire was used to collect data from the adolescents at school. All information was gathered from the adolescents, except for parental education which was reported by parents themselves as part of the informed consent for the adolescent.

#### Outcome behaviors

Consumption of vegetables was assessed by frequency questions with categories ranging from never/seldom to three times per day or more. Separate questions were used to assess the consumption of raw and cooked vegetables. The frequency of total weekly vegetable consumption (sum of raw and cooked vegetables) was computed. Participants were informed that they should not include potato consumption. These questions have previously been validated among 11-year-olds with a 7-day food record as the reference method, and were found to have a satisfactory ability to rank subjects according to their intake [26].

Intake of carbonated sugar-sweetened soft drinks (hereafter referred to as soft drinks) was assessed separately for weekdays and weekend days. For weekdays, consumption was assessed using a frequency question (with categories ranging from never/seldom to every weekday) and amount in glasses each time soft drink was consumed (from one glass to four glasses or more). Frequency and amount in glasses were multiplied to get a weekday consumption measure in glasses which was then converted into deciliters. For weekends, only amount in glasses consumed on both days was asked (eight categories: from never/seldom to seven glasses or more) and was converted into deciliters. Since soft drinks are often sold in 500 ml bottles or cans, participants were informed that ½ liter = 3 glasses (therefore one glass = 1.67 dl). Weekday and weekend intakes were summed up to create a weekly intake variable. The questions assessing the intake of soft drinks have been validated among 9- and 13-year-old Norwegians using a 4-day precoded food diary as the reference method, and moderate correlation coefficients were obtained [27].

Moderate to good test-retest reliability (ICC = 0.58–0.78) has previously been obtained for the measures of both dietary behaviors [25].

#### Perceived accessibility and perceived parental rules

Perceived accessibility of vegetables was assessed using a scale with four items and perceived accessibility of soft drinks was assessed using a scale with three items. Perceived rules related to the consumption of vegetables was assessed using a scale with two items and perceived parental rules for the consumption of soft drinks was assessed using a scale with four items.

A 5-point Likert scale with answer categories ranging from “totally disagree to totally agree” was used for all these variables. Table 2 shows the details of the items included.

The items used in the scales were developed based on existing literature [28–30] as well as feedbacks provided by an expert group. One of the aims was to explore whether adolescents would include perceived rules as part of their report of accessibility or whether perceived rules and perceived accessibility are separate correlates/factors. The expert group included five professors, four postdoctoral researchers and one lecturer with different backgrounds related to family processes and dietary habits (nutrition, behavioral sciences, nursing, clinical nutrition, public health, psychology and health promotion). This group also assessed the face and content validity of the scales used. A pre-test study using cognitive interviews was conducted among five 13 year-olds (two boys, three girls) to ensure that they understood the items and response scales. Minor changes were made in wording. Thereafter, a pilot study including a time test and a written evaluation of the full questionnaire was conducted among 17 adolescents (nine girls, eight boys). The questionnaire was completed in about 25–45 min (average 36 min).

Details of the procedures followed in the development of the scales have already been published [25].

#### Sociodemographic measures

Parental education was categorized into low (12 years of education or less, which corresponded to secondary education or lower) and high (13 years of education and more, which corresponded to university or college attendance). Educational status of the parent with the longest education or else the one available was used in the analyses.

Participants were divided into either ethnic Norwegian or ethnic minority. Ethnic minorities were defined as those having both parents born in a country other than Norway [31].

### Statistical analyses

Descriptive analyses were first conducted. Principal component analysis with Varimax rotation was conducted on the items assessing accessibility and rules. An item was identified to load on a given component if the factor loading was >0.60 and had no cross-loadings >0.5 on any other factor [32]. The number of factors was based on eigenvalues (>1). Intra-class correlation coefficients (ICC) were used to assess the test-retest reliability and classified as follows: “excellent” (≥0.81), “good” (0.61–0.80), “moderate” (0.41–0.60), ‘poor’ (≤0.40) [33]. Corrected Item-Total Correlation (CITC) and Cronbach’s alpha were used to assess the internal reliability of the construct. CITC >0.30 were considered good, and <0.15 were considered unreliable [34]. Cronbach’s alpha was classified as α < 0.5 = unacceptable, 0.5 ≤ α < 0.6 = poor, 0.6 ≤ α < 0.7 = acceptable and 0.7 ≤ α < 0.9 = good [35].

**Fig. 1:**
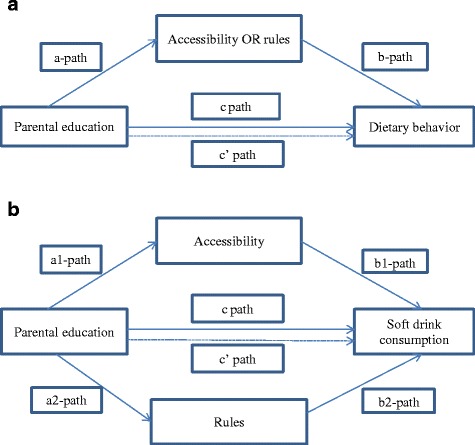
Single and multiple mediation models

Single mediation analyses were then conducted. Figure 1a depicts the single mediation model followed. In a single mediation, the a-path represents the association between parental education and the mediator. The b-path represents the association between the mediator and the relevant dietary behavior adjusted for parental education. The c’ path represents the association between education and the dietary behavior (adjusted for the mediator and covariates). As rules related to the consumption of vegetables were not found to have a significant mediating role, multiple mediation analysis was conducted only for soft drink consumption. Figure 1b shows the multiple mediation model used. In the multiple mediation analysis, the a1-path represents the association between parental education and the first mediator and the a2-path represents the relationship between parental education and the second mediator. The b-paths represent the association between the first mediator (b1-path) and the second mediator (b2-path) and the consumption of soft drinks (adjusted for parental education and covariates). The c’ path represents the association between education and the consumption of soft drinks when adjusted for both mediators and covariates. Bootstrap corrected CIs were calculated for indirect effects (a*b). Bootstrapping (1000 samples) was conducted using the PROCESS macro for SPSS by Andrew Hayes [36]. The percentage mediated was also reported.

## Results

Table 1 presents the characteristics of participants. The mean age of the adolescents was 14.3 years (SD = 0.6) and 52 % were girls. In total 34 % of the adolescents had parents with low level of education (<12 years). In the total sample, 91 % of participants were ethnic Norwegian; among those with low parental education, the respective proportion was 83 %. The adolescents’ mean intake of soft drinks was 690 ml/week (C.I. 620–760), and the frequency of vegetable intake was 9.5 times per week (C.I. 8.6–10.0).

**Table 1:** Characteristics of study participants (*n* = 440), results are presented as percentages or means (CI)

Characteristics of participants	Total sample	Low parental education (*n* = 141)^a^	High parental education (*n* = 276)^a^	*p* value*
Age (years)	14.3 (14.2–14.3)	14.5 (14.4–14.6)	14.3 (14.2–14.3)	0.003
Gender (% girls)	52.3	53.9	51.4	0.60
Ethnicity (% ethnic Norwegian)^b^	90.9	82.9	95.3	<0.001
Intake of soft drinks (ml/wk)	680 (620–760)	840 (690–980)	600 (530–680)	0.004
Intake of vegetables (times/wk)	9.4 (8.8–10.0)	8.5 (7.4–9.5)	10.1 (9.3–10.9)	0.021
Perceived accessibility of soft drinks^c^	2.40 (2.29–2.51)	2.65 (2.48–2.84)	2.25 (2.10–2.39)	0.001
Perceived rules related to the consumption of soft drinks^c^	3.95 (3.83–4.07)	3.68 (3.48–3.89)	4.08 (3.92–4.23)	0.002
Perceived accessibility of vegetables^c^	4.02 (3.94–4.10)	3.84 (3.70–3.98)	4.10 (4.00–4.19)	0.001
Perceived rules related to the consumption of vegetables^c^	4.47 (4.39–4.56)	4.43 (4.28–4.59)	4.50 (4.40–4.60)	0.50

^*^ *P* value for the difference between parental education groups using *t*-test and chi-squared tests

^a^ Low parental education: 12 years of education or less, high parental education: 13 years of education and more

^b^ Ethnic minorities are defined as those having both parents born in a country other than Norway

^c^ Measured using a 5-point Likert scale

Factor analyses confirmed two separate subscales, named “accessibility” and “rules”, both for vegetables and soft drinks. The factor loading for all items was >0.60 (Table 2). The values of CITC were good (>0.50) for all of the items (Table 3). The Cronbach’s alpha values for the scales assessing perceived rules and perceived accessibility of vegetables were 0.79 and 0.76 respectively. The Cronbach’s alpha values for the scales assessing perceived rules and perceived accessibility of soft drinks were 0.70 and 0.87 respectively. The test–retest reliability of the scales was moderate to good (0.44–0.62) (Table 3).

**Table 2:** Factor loading of scales assessing perceived rules and accessibility of vegetables and soft drinks (*n* = 440)

Vegetables	Factor 1	Factor 2
At home..	*n* = 431	*n* = 435
.. we vary the types of vegetables served for dinner during a week	0.831	
.. we vary how the vegetables are prepared for dinner (raw, cooked etc.) during a week	0.789	
.. we usually have vegetables for dinner every day	0.697	
.. there are usually vegetables that I like available	0.628	
.. I can eat vegetables whenever I want		0.891
.. I can eat as many vegetables as I want to		0.887
Eigenvalues	2.42	1.71
Proportion of variance	34.63	24.41
Soft drinks	Factor 1	Factor 2
At home.. (completely agree to completely disagree)	*n* = 436	*n* = 437
.. we usually have soft drinks for dinner on weekend days	0.824	
.. there are usually soft drinks available	0.776	
.. we usually have soft drinks for dinner on week days	0.698	
.. we have rules for when I can drink soft drinks (reverse coded)		0.900
.. we have rules for how much soft drinks I can drink (reverse coded)		0.828
.. I can drink soft drinks whenever I want		0.733
.. I can drink as much soft drinks as I want to		0.718
Eigenvalues	2.26	2.68
Proportion of variance	32.29	38.26

**Table 3:** Psychometric properties of scales assessing perceived rules and accessibility of vegetables and soft drinks (*n* = 440)

Vegetables	Mean	SD	CITC^a^	α^b^	ICC^c^
Total score, accessibility – dinner (range: 1–5)	4.02	0.81		0.76	0.51
At home..					
..we usually have vegetables for dinner every day	4.24	0.97	0.52		
.. we vary the types of vegetables served for dinner during a week	3.97	1.04	0.64		
.. we vary how the vegetables are prepared for dinner (raw, cooked etc.) during a week	3.82	1.13	0.55		
.. there are usually vegtetables that I like available	4.09	1.10	0.50		
Total score, rules (range: 1–5)	4.47	0.86		0.79	0.51
At home..					
.. I can eat vegetables whenever I want	4.44	0.98	0.66		
.. I can eat as many vegetables I want to	4.49	0.91	0.66		
Soft drinks	Mean	SD	CITC	α	ICC
Total score, accessibility – dinner (range: 0–5)	2.40	1.18		0.70	0.62
At home..					
.. there are usually soft drinks available	2.63	1.63	0.56		
.. we usually have soft drinks for dinner at week days	1.30	1.03	0.50		
.. we usually have soft drinks for dinner at weekend days	3.32	1.72	0.57		
Total score, rules (range: 0–5)	3.95	1.23		0.87	0.44
At home..					
.. we have rules for when I can drink soft drinks	3.87	1.57	0.74		
.. we have rules for how much soft drinks I can drink	3.57	1.57	0.65		
.. I can drink soft drinks whenever I want	4.14	1.38	0.75		
.. I can drink as much soft drinks as I want to	4.22	1.32	0.74		

^a^ Corrected Item-Total Correlation for assessment of internal reliability

^b^ Chronbach’s alpha for assessment of internal reliability

^c^ Intra-class correlation assessing test-retest reliability

As shown in the c-path in Table 4, there was a significant positive association between parental education and the consumption of vegetables, although group differences were not large. There was also a significant inverse association between parental education and the consumption of soft drinks after adjusting for gender and ethnic background.

**Table 4:** Mediating effects of perceived rules and accessibility in the relationship between parental education and the consumption of vegetables and soft drinks (*n* = 417)

	c-path	c’-path	a-path	b-path	a*b	% mediated
Single mediation models						
Vegetable (times/wk)						
Perceived rules	**1.991 (0.680, 3.301)**	**1.674 (0.349, 2.999)**	0.071 (−0.108, 0.250)	**1.238 (0.518, 1.958)**	0.088 (−0.137, 0.371)	
Perceived accessibility		0.975 (−0.215,2.166)	**0.290 (0.123, 0.458)**	**3.495 (2.807, 4.182)**	**1.015 (0.411, 1.710)**	51.0
Soft drinks (dl/wk)						
Perceived rules	**−2.581 (−4.061, −1.101)**	**−1.928** (−**3.351, −0.504)**	**0.371 (0.111, 0.631)**	**−1.759 (−2.293, −1,224)**	**−0.653 (−1.342, −0.247)**	25.3
Perceived accessibility		−1.224 (−2.555, 0.106)	**−0.459 (−0.698, −0.220)**	**2.958 (2.419, 3.550)**	**−1.358 (−2.379, −0.725)**	52.6
Multiple mediation model						
Soft drinks (dl/wk)						
Perceived rules	**−2.581 (−4.061, −1.101)**	−1.125 (−2.455, 0.206)	**0.364 (0.102, 0.625)**	**−0.597 (−1.160, −0.033)**	**−0.217 (−0.671, −0.017)**	8.5
Perceived accessibility			**−0.458 (−0.699, −0.218)**	**2.645 (2.032, 3.259)**	**−1.212 (−2.012, −0.592)**	47.5
Total					**−1.429 (−2.344, −0.730)**	56.0

Significant values are shown in bold

All paths are adjusted for gender and ethnicity

As shown in the a-path in Table 4, parental education was positively associated with accessibility of vegetables and rules related to the consumption of soft drinks and inversely related to the accessibility of soft drinks. There was no significant association between parental education and rules related to the intake of vegetables. Single mediation analyses further showed that accessibility was a significant mediator in the association between parental education and the consumption of vegetables and mediated 51 % of the total effect. No mediating effect was found for rules related to the consumption of vegetables. In single mediation analysis, accessibility and rules were found to be significant mediators of the association between education and the consumption of soft drinks (52.6 and 25.3 % of total effect mediated respectively). In multiple mediation analyses, both correlates remained significant mediators but the percentage of total effect mediated changed to 47.5 and 8.5 respectively. The c’-path (direct effect) was no longer significant.

## Discussion

The first aim of the present study was to assess the psychometric properties of new scales assessing the perceived accessibility and rules related to soft drinks and vegetable consumption. The second aim was to explore the mediating effect of these correlates in the relationship between parental education and the respective dietary behaviors. Exploratory factor analyses confirmed the presence of four factors: two reflecting the accessibility of soft drinks and vegetables and two reflecting rules related to these dietary behaviors. The scales measuring these factors were found to have adequate test-retest and internal consistency reliability. Accessibility was found to be a significant mediator of the association between parental education and both dietary behaviors. Rules related to the consumption of soft drinks mediated the association between parental education and soft drink consumption.

Accessibility reflects whether foods are available in a form and location that facilitate their consumption [37]. In Norway, vegetables are eaten mostly as part of dinner which is typically the only hot meal eaten during the day; 70 % of the consumption of vegetables occurs during dinner [38]. Breakfast and lunch are typically made up of cold meals containing breads or cereals, and consumption of vegetables is limited during these meals. Soft drinks are often served for dinner during weekend days. Therefore, the measures of correlates used in this study focused on accessibility for dinner. For vegetables, variation in type and variation in preparation were further added as part of increased accessibility. Rules regarding how much and how often the food items could be consumed were also assessed, and were specific to the dietary behaviors included. Previous studies have assessed one or more measurement properties of scales assessing similar constructs among children [18, 39–43]. Direct comparison between studies is however not possible as the number and content of items used to assess these constructs have varied between studies. However, the findings of adequate internal consistency and test-retest reliability in this study are generally comparable to the findings of these studies [18, 39–43], except for test-retest reliability which was low for a measure of accessibility among 4–6^th^ graders [42]. Most of the aforementioned studies included participants younger than those in the present study. The reliability and validity of self-reported measures might vary between children and adolescents [44]. Adolescents are likely to be more independent than children with regards to when and where they consume food; they are also more likely to be influenced by peers [45, 46]. This might affect results of validity and reliability of measures such as accessibility of food at home. Therefore it is important to test measures separately among children and adolescents. In four of the six aforementioned studies, single item measures were used to assess accessibility or rules related to vegetable or soft drink consumption. However, single items are unlikely to capture the multiple dimensions of rules and accessibility [47]. The new scales developed in the present study therefore represent a more comprehensive measurement of these constructs. In addition, the measures of accessibility were adapted to the Norwegian food consumption culture (i.e. focus on dinner). However, the focus on dinner for the assessment of accessibility might imply that the measures would be most useful in contexts where dinner constitutes an important source of vegetable and soft drink consumption.

The important role of accessibility for the dietary behaviors of youth is well known [5–7, 9], and the present study confirms these findings. Accessibility of soft drinks was significantly higher among those with low parental education, in line with findings of other studies [9, 20, 21]. Accessibility was a significant mediator of the relationship between parental education and soft drink consumption. This mediating role of accessibility was previously found in a study among Norwegian 11–13 year-olds [20]. The conceptualization of accessibility in that study was, however, different from the present study, as participants were asked whether they can serve themselves as they please when soft drinks are available. Among young children, soft drink served at meals was found to be a significant mediator in the association between maternal education and soft drink consumption, accounting for 51 % of the mediating effect [9]. In the present study, accessibility of soft drinks was not only higher among those with low parental education, it was also a stronger predictor of soft drink intake in this group (significant parental education*accessibility interaction effect, results not shown). These factors explain its important mediating role. Children with parents with high education reported stronger rules prohibiting the consumption of soft drinks, as reported in previous studies [16, 48]. In the multiple mediation model however, the role of rules as a mediator in the association between parental education and the consumption of soft drinks was significant but relatively weak, with only 8 % of the total effect mediated by rules. Physical factors such as accessibility therefore appear more important in explaining differences in soft drink consumption between adolescents having parents with high and low parental education than the psychosocial environment.

Perceived accessibility of vegetables was lower among those with low parental education as reported in previous studies [16]. Accessibility was also a significant mediator with 51 % of the total effect mediated by it. One previous study identified accessibility as the strongest mediator in the relationship between parental education and FV consumption, which is supported by the findings of the present study for vegetable consumption [22]. An increase in disparity in accessibility was also found to partly mediate an increasing SEP disparity in FV consumption among adolescents over a 7-year period [19]. It has been suggested that, in settings where availability of FV is likely higher (high SEP), accessibility might be less important than when availability is lower [16]. Availability is a prerequisite for accessibility and might by itself have an effect on intake and on socioeconomic differences [9, 20]. However, the findings of this study indicate that increasing accessibility by serving vegetables for dinner and by varying the type and preparation might be particularly important.

The results of the present study are important for future interventions aimed at increasing vegetable consumption and decreasing the intake of sugar-sweetened beverages while addressing socioeconomic differences as they give an indication about intervention components to focus on. The findings particularly highlight the role of soft drink and vegetable accessibility during dinner. It has been suggested that focusing on situational behavior (e.g. consumption at dinner) would allow for a better explanation of dietary behaviors which are complex and difficullt to change [49]. Similarly, focusing on behavior specific and situation specific correlates of dietary behaviors might allow for more efficient interventions targeting these correlates. Dinner is often a family meal and parents can have a good control of what is served and what is consumed. Therefore it is important to encourage and educate parents, in particular those with low socioeconomic position, to increase accessibility of vegetables, as well as decrease accessibility of soft drinks during dinner. Encouraging the reinforcement of rules limiting soft drink intake might also play some role in reducing socioeconomic differences in soft drink consumption. These efforts could be achieved through different public health initiatives. The use of established systems which can allow access to parents from all socioeconomic backgrounds might particularly be important as it will provide an opportunity for reaching a significant number of youth. These systems include the health services such as child health clinics which in Norway provide universal health care, as well as school nurses who can have contact with all parents during parental meetings etc. Further research looking at other individual and home environmental mediators (e.g. nutrition knowledge, parental modeling) of socioeconomic differences in the dietary behaviors included is also warranted. Research including broader environmental (i.e. school and neighborhood environmental) determinants is particularly needed. Whether and how ethnic differences in parental education contribute to the parental educational differences in the dietary behaviors should also be explored.

### Strengths and limitations

The results of this study should be seen in light of the following weaknesses. The cross-sectional data used in the study does not allow for any inference about causality. Ideally, mediation analyses should be conducted using longitudinal data. However, when exploring the association between parental education and the dietary behaviors and mediators included, it is likely that the directions of associations are accurate (parental education would influence these factors and not vice versa). The socioeconomic differences in vegetable consumption were not large. This could, among other things, be due to the way intake was measured (using categories and mid-points for some categories). Another reason could be the rather broad categorization of educational groups. The use of self-report measures of dietary behaviors and mediators is another weakness of the present study as such measures are liable to recall and social desirability bias. However, adequate test-retest reliability was obtained for these measures. The participation rate was also fairly low, in particular the participation rate in the test-retest study. It was not possible to ask participants their reasons for non-participation. It was also not possible to compare adolescents who participated in the study and those who did not, since there was no available information regarding non-participants. The test-retest was conducted at one school in a high socioeconomic area; therefore the generalizability of the test-retest results might be limited to those with high SEP. The sample size for the test-retest reliability analyses was also fairly low; it therefore appears useful to further assess the test-retest reliability of the scales using larger samples. Education was the only indicator of SEP included in the present study. It was reported by parents themselves which was a strength as it allowed for fairly complete data; parental reports of education are also likely to be more reliable than adolescent reports. Parental education is also the socioeconomic indicator found to be most commonly associated with dietary behaviors and their correlates among youth [16], as well as with body weight [50]. There is an association between education and other indicators of SEP such as income and occupation in Norway [51]. Although education, income and occupation are correlated, existing research suggests that the association between these indicators of SEP and dietary behaviors among children is specific/independent [52]. Therefore, including measures of income and occupation in future studies might allow for the exploration of the independent effects of these indicators on dietary behaviors. Other demographic variables such as parental age and family structure should also be considered in future studies. Finally, although more comprehensive compared to single item measures, multiple item scales increase participant burden as they require more time to complete, which needs to be taken into consideration when designing questionnaires including such scales.

## Conclusion

The new scales developed in this study provide a more comprehensive assessment of parental rules and accessibility among adolescents than several existing measures of the same constructs; the scales assessing accessibility were also adapted to the local food consumption patterns. The scales have adequate evidence of validity and reliability and are therefore considered appropriate for use in surveys among adolescents. A significant proportion of the parental educational differences in soft drink and vegetable consumption was explained by differences in accessibility of soft drinks and vegetables during dinner. Therefore, decreasing accessibility of soft drinks and increasing accessibility of vegetables during dinner is important for all groups and particularly for those with low parental education.

## Abbreviations

CITC: Corrected Item-Total Correlation
FV: Fruit and Vegetables
ICC: Intra-Class Correlation
SEP: Socioeconomic Position
